# Design and process of the EMA Cohort Study: the value of antenatal education in childbirth and breastfeeding

**DOI:** 10.1186/1472-6955-7-5

**Published:** 2008-04-24

**Authors:** Carmen Paz-Pascual, Isabel Artieta Pinedo, Gonzalo Grandes, Gurutze Remiro Fernandez de Gamboa, Itziar Odriozola Hermosilla, Amaia Bacigalupe de la Hera, Janire Payo Gordon, Guadalupe Manzano Garcia, Magdalena Ureta de Pedro

**Affiliations:** 1 Sestao Health Centre – Ezkerraldea Enkarterri district, Basque Health Service (Osakidetza). Spain. Midwife Teaching Unit of the Basque Country, Biscay, Spain; 2 Barakaldo Health Centre – Ezkerraldea Enkarterri district, Basque Health Service (Osakidetza). Spain; 3 Primary Care Research Unit, Basque Health Service (Osakidetza) Bilbao, Biscay, Spain; 4 Cruces Hospital, Basque Health Service (Osakidetza), Barakaldo, Spain; 5 Basurto Hospital, Basque Health Service (Osakidetza), Bilbao, Spain; 6 La Rioja University, La Rioja, Spain; 7 Sestao Health Centre – Ezkerraldea Enkarterri district, Basque Health Service (Osakidetza), Spain

## Abstract

**Background:**

Antenatal education (AE) started more than 30 years ago with the purpose of decreasing pain during childbirth. Epidural anaesthesia has achieved this objective, and the value of AE is therefore currently questioned. This article describes the protocol and process of a study designed to assess AE results today.

**Methods/Design:**

A prospective study was designed in which a cohort of 616 nulliparous pregnant women attending midwife offices of the Basque Health Service were followed for 13 months. Three exposure groups were considered based on the number of AE sessions attended: (a) women attending no session, (b) women attending 1 to 4, and (c) women attending 5 or more sessions. Sociodemographic, personality, and outcome variables related to childbirth and breastfeeding were measured.

It was expected 40% of pregnant women not to have participated in any AE session. However, 93% had attended at least one session. This low exposure variability decreased statistical power of the study as compared to the initially planned power. Despite this, there was a greater than 80% power for detecting as significant differences between exposure groups of, for instance, 10% in continuation of breastfeeding at one and a half months and in visits for false labour. Women attending more sessions were seen to have a mean higher age and educational level, and to belong to a higher socioeconomic group (p < 0.01). Follow-up was completed in 99% of participants.

**Discussion:**

Adequate prior estimation of variability in the exposure under study is essential for designing cohort studies. Sociodemographic characteristics may play a confounding role in studies assessing AE and should be controlled in design and analyses. Quality control during the study process and continued collaboration from both public system midwives and eligible pregnant women resulted in a negligible loss rate.

## Background

There are currently serious doubts about the value of antenatal education (AE) in childbirth outcome and start of breastfeeding [1,2]. The current AE model was designed in the 60's and was clearly aimed at decreasing pain during delivery, an objective achieved today by epidural anaesthesia, that is widely used in our practice. For instance, epidural anaesthesia is used in more than 97% of deliveries in centres from the Basque Health Service in Biscay [3]. There is therefore a need for adapting current objectives of AE to the new needs and demands of the population.

AE is associated to shorter dilation and expulsion periods [4-6], a decreased demand for anaesthesia [7], or a higher rate of start of breastfeeding [8]. However, it has also been related to adverse results such as longer expulsion periods and a higher rate of instrumental deliveries [9]. At any rate, the effects are not conclusive [10,2].

The purpose of this research protocol was to assess the effect of attendance to AE sessions on childbirth outcome and on the start and continuation of breastfeeding during the first year.

This research project will promote understanding of the effect of AE within the current reference context and will allow for improving AE by adapting it to the current needs of pregnant women.

## Methods/Design

A prospective, observational, longitudinal study was designed to follow up a cohort of pregnant women from the Biscay health area attending midwife offices of the public Basque Health Service/Osakidetza, in order to assess the effect of AE exposure on childbirth and breastfeeding. The project was approved by the Committee for Primary Care Research of the Basque Health Service/Osakidetza.

### Study sites

Thirty-four centres, 65% of those having primary care midwives within the Basque Health Service/Osakidetza, collaborated in the project. All four districts of the Biscay health area were represented.

AE is given in all collaborating primary care centres. This consists of at least 8 sessions where breathing techniques, pushing, and relaxation are practised, and different topics such as labour and delivery, postpartum period, newborn care, and breastfeeding are addressed. This study assumes that, in our area, AE sessions are relatively similar in terms of structure, duration, topics, and body work practised, while recognising that every healthcare professional may make use of his/her specific knowledge and pay special interest to any particular aspect.

The two hospitals of the public Basque Health Service having a delivery area (Hospital de Cruces and Hospital de Basurto) collaborated in the study.

### Participants

All nulliparous pregnant women from the Biscay health area aged 18 to 42 years who attended the office of a primary care midwife from week 36 of pregnancy and who did not meet any exclusion criterion (Table 1) were eligible for the study.

**Table 1:** Exclusion criteria for participants in the EMA study

**Multiple pregnancy**
**Pathological pregnancy**
· Clinically and/or radiographically documented pelvic abnormality
· Prior uterine malformation
· Uterine tumour in current pregnancy
· Prior uterine surgery
· Prior genital tract abnormality
· Positive cytologic testing for malignant cells
· Prior severe medical or surgical disease (maternal heart disease restricting physical capacity of the woman, neuropathy, coagulopathy, diabetes, etc.)
· Late cerclage, performed after 16 weeks
· Active toxoplasmosis during pregnancy
· German measles during pregnancy
· Sexually transmitted infection during current pregnancy
· Gestational diabetes
· Rh isoimmunisation
· Foetal malformation
· Placenta previa
· Pregnancy-induced HBP
· Hydramnios of 2 or more litres of amniotic fluid
· Oligohydramnios lower than 500 mL.
· Bleeding in the third trimester
· Suspected or documented IUGR
· Threatened premature birth
Uncontrolled pregnancy*
**Difficult follow-up**
· No telephone, fixed abode, and others
**Not speaking Spanish or Basque**

Adequate pregnancy control: 6 obstetric visits, some of them in the first trimester of pregnancy. (SEGO, 2002).

Recruitment lasted eight months, from September 2005 to May 2006. Primary care midwives invited a consecutive sample of nulliparous pregnant women eligible to participate to collaborate in the study. All women collaborating in the study signed an informed consent explaining the main study aspects and collaboration conditions.

Thirty primary care midwives collaborated in the study by proposing participation to all pregnant women eligible for inclusion, discussing with them the whole study process, providing informed consent, and collaborating in the initial measurements.

Hospital care midwives and resident midwives at both Hospital de Cruces and Hospital de Basurto detected pregnant women in the study and performed measurements during the labour, delivery and postpartum periods at the hospital.

### Measurements

AE exposure was measured by counting the AE sessions attended by each participant. This variable was measured during the postpartum period by hospital midwives and for incomplete records, by telephone interview conducted one and a half months after childbirth. Three exposure groups were formed based on the number of sessions attended by participants: (A) women attending no session, (B) women attending 1 to 4 sessions, and (B) women attending 5 or more sessions. Sixty percent of nulliparous pregnant women were expected to attend any AE session, and no AE exposure was expected in the remaining 40%.

Potential confounding variables recorded included age, sociodemographic characteristics such as social class, educational level and nationality [11], and woman personality, that may influence both AE session attendance or otherwise and childbirth outcome and breastfeeding. The personality measurement instrument was the Battery of scales of generalized expectancies for control (BEEG-20) [12].

Outcome variables related to objective childbirth aspects that were measured included: (1) number of visits for false labour (visits to the emergency room in a latent labour stage not leading to admission for labour), (2) duration of dilation stage in minutes, (3) duration of expulsion stage in minutes, (4) cervical dilation in centimetres at the time epidural anaesthesia is administered, (5) type of delivery (normal/instrumental/Caesarean section), (6) presence of episiotomy and/or tear and grade [13], (7) sex of the newborn (male/female), (8) weight of the newborn in grams, and (9) Apgar test of the newborn. Objective childbirth variables were collected from the clinical records of pregnant women.

Subjective childbirth variables measured included: (10) degree of anxiety according to the Hospital Anxiety and Depression Scale (HAD) [14], and (11) degree of satisfaction caused by this life experience, assessed based on the answers to two questions asked after childbirth: (a) How would you rate your childbirth experience in a scale where 0 would be very negative and 10 very positive? And (b) In relation to the expectations you had, would you say that your childbirth experience has been: Better than you expected/As you expected/Worse than you expected? The HAD anxiety scale was measured during the dilation period, always before the start of the expulsion period and satisfaction once delivery had ended.

Breastfeeding variables measured included: (12) early start (within 2 hours of childbirth) and (13) continuation of breastfeeding, either alone or combined with other feeding, at one and a half, three, six, nine, and 12 months of life. Early start of breastfeeding was recorded by hospital midwives and continuation of breastfeeding was recorded through telephone surveys conducted from the Primary Care Research Unit.

### Statistical analysis

The estimated sample size was 657 women. Assuming that 40% of these women did not attend AE, this size provided a statistical power greater than 95% for detecting as significant 15% differences in false labour, anaesthesia in latent stage, instrumental deliveries and breastfeeding after one and a half months, and 20% differences in anxiety and episiotomy, and for detecting equivalence in the duration of the dilation and expulsion periods. Alpha error level is less than 5%.

The basic analysis will involve the calculation and comparison of proportion of dichotomous outcomes and averages of quantitative results among the cohorts exposed to different levels of AE. Both relative and absolute measures of association with 95% confidence intervals will be calculated and tested by chi-square and t tests The exposure groups will also be compared to detect unbalances with respect to potential confounding variables and characteristics that could be associated with the outcomes under study. To control for the eventual confounding effect of those characteristics, stratified analyses and multivariate statistical models will be used. These models will be linear for continuous outcomes an logistic for dichotomous ones. These same models will be extended to mixed effects models in which the midwife will be included as a random effect to take into consideration the hyerarchical structure of data (pregnant women clustered in the practice of each midwife) and to estimate the particular effect of each of them on the results. To analyse time to discontinuation of breastfeeding, Cox proportional hazards models will be adjusted. All analyses will be performed using EpiInfo (Centers for Disease Control and Prevention -CDC- V. 3.3.2 2005) and SAS (SAS Institute Inc., Cary NC, 2003) softwares.

### Study process

**Figure 1:**
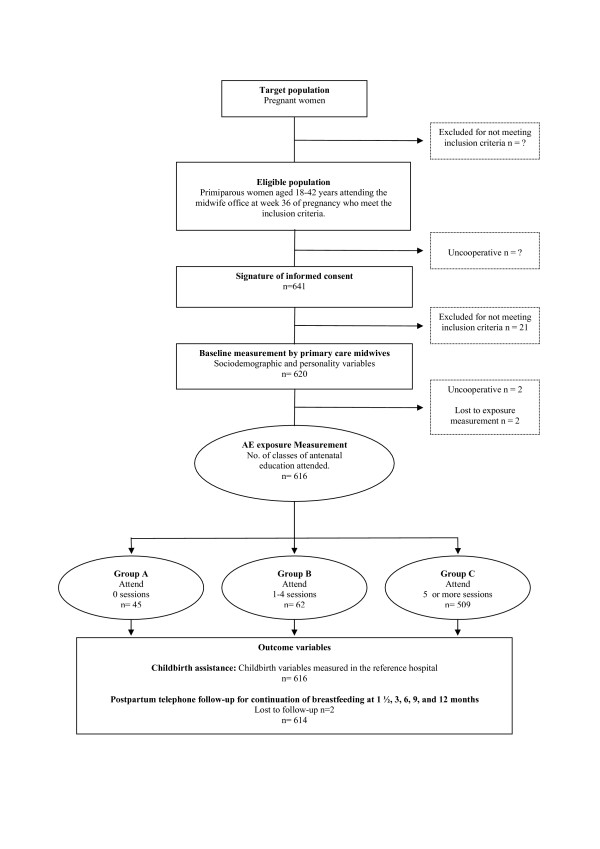
Flowchart of the cohort study EMA.

The number of eligible women who refused to participate in the study was very low according to midwives in charge of recruitment, but was not recorded. As shown by the study flowchart (Figure 1), 641 women eligible to participate in the study were recruited from September 2005 to May 2006. Among these 641 participants, 21 were withdrawn from the study because they met some initially undetected exclusion criterion. Finally, 620 pregnant women were included in the EMA study. Study losses included two women who did not have their babies in the reference hospitals and two women who refused to continue collaborating with the study. Thus, 616 women finally participated in the study. Breastfeeding measurements were performed in all women but two (99%).

Forty percent of pregnant women were expected not to have participated in any AE session. However, the proportion of pregnant women who did not attend AE did not exceed 7.3% of participants despite prolongation of the recruitment period from 4 to 8 months, while 92.6% of nulliparous pregnant women attended AE. This low exposure variability decreased statistical power of the study as compared to the initially planned power. Despite this, there was a greater than 80% power for detecting as significant differences between exposure groups, as shown in Table 2.

**Table 2:** Hypothesised differences between antenatal education exposure groups for whose detection the EMA study has a statistical power greater than 80%

N = 616	**Group A **(0 sessions) n = 45 (7.3)	**Group B **(1–4 sessions) n = 62 (10.1)	**Group C **(≥ 5 sessions) n = 509 (82.6)
Visiting hospital for false labour	31%	22%	14%
Anxiety: HAD scale	55%	48%	35%
Mean duration of dilation. (SD 201 min)	470	425	375
Mean duration of expulsion period (SD 68 min)	100	85	70
Dilation in epidural anaesthesia. (SD 1.28 cm.)	3.4	3.7	4
Anaesthesia administered in latent birth stage	39%	30%	20%
Instrumental deliveries	30%	20%	13%
Episiotomy in normal births	55%	48%	35%
Breastfeeding at one and a half months	55%	64%	75%
Breastfeeding at 3 months	48%	58%	69%
Breastfeeding at 6 months	24%	35%	47%

Mean age of participants was 31.3 years, virtually all of them are Spanish in nationality (94.3%), and a great majority have a secondary school or university educational level (n = 510). With regard to comparability of exposure groups, sociodemographic characteristics of pregnant women are seen to differ depending on grade of AE exposure. Thus, women attending more sessions (group C) are older (p < 0.01), have a higher educational level (p < 0.01) and socioeconomic level (p < 0.01), and are Spanish in a greater proportion. As to personality characteristics, pregnant women in all three groups (A, B, and C) are similar in all personality scales (Table 3).

**Table 3:** Sociodemographic and personality characteristics of participants in the ema study

**Variable**	**Group A **(0 sessions) n = 45 (7.3)	**Group B **(1–4 sessions) n = 62 (10.1)	**Group C **(≥ 5 sessions) n = 509 (82.6)	**TOTAL **n = 616
Mean age (SD)*	28.7 (4.7)	30 (4.5)	31.6 (4)	31.2 (4.2)
Nationality*				
Spanish	38 (84.4)	57 (91.9)	486 (95.5)	581 (94.3)
Others	7 (15.6)	5 (8.1)	23 (4.5)	35 (5.7)
Educational level*				
No schooling	0 (0)	0 (0)	1 (0.2)	1 (0.2)
Primary education	5 (11.1)	7 (11.3)	5 (1)	17 (2.8)
Lower examination	14 (31.1)	8 (12.9)	66 (13)	88 (14.3)
Higher certificate	18 (40)	29 (46.8)	226 (44.4)	273 (44.3)
University	8 (17.8)	18 (29)	211 (41.5)	237 (38.5)
Social class*				
Low	31 (68.9)	28 (45.2)	216 (42.4)	275 (44.6)
Middle	8 (17.8)	22 (35.5)	155 (30.5)	185 (30)
High	6 (13.3)	12 (19.4)	138 (27.1)	156 (25.3)
**Battery of Scales of Generalized Expectancies for Control: BEEGC**				
Contingency, mean (SD)	30.8 (4.4)	31.1 (3.5)	30.2 (4.0)	30.3 (4)
Helplessness, mean (SD)	14.2 (7.2)	12.6 (5.9)	12.2 (5.8)	12.4 (5.9)
Luck, mean (SD)	18.7 (7.6)	16.4 (6.5)	16.5 (6.4)	16.6 (6.5)
Self-efficacy, mean (SD)	27.4 (3.8)	27.3 (4.9)	26.6 (4.1)	26.7 (4.2)
Success, mean (SD)	29.2 (4.7)	29.9 (4.4)	29.1 (4.0)	29.2 (4.1)

Values are numbers (percentages) of women, unless otherwise specified. n = 616.

* Significant differences (p < 0.01).

### Quality control

During data collection in and after childbirth, two specially designated midwives, one at each hospital, monitored measurements to minimize potential interferences or losses.

The whole process, including recruitment and measurements, was supervised and controlled by the Primary Care Research Unit of Biscay. Indicators used for controlling such processes were as follows:

(a) weekly recruitment rate: 2 women per week each midwife

(b) percent losses during the process: less than 7%

(c) proportion of inadequately completed forms: less than 2%

## Discussion

When designing cohort studies such as the one reported here, it is very important to adequately anticipate the expected variability in the exposure under study. In this case, the expected variability when the study was designed was greater than the actual variability. As noted above, 40% of pregnant women were not expected to attend AE, but only 7% did not actually attend AE. Study restriction to nulliparous pregnant women markedly decreased the possibility of recruiting women who do not attend AE because of the high demand for this activity among first time mothers. However, such restriction allowed for more clearly delineating the effect of AE by removing the effect of prior birth experiences and antenatal education. This imbalanced variability in AE exposure is a disadvantage also found by other European studies [1]. In our case, a decrease in study power as compared to the initially planned power had to be assumed.

Sociodemographic characteristics of participants were different in the three comparison groups A, B, and C. This suggests a potential confounding role of such characteristics, as they may influence both attendance to AE sessions and the outcome of birth and breastfeeding. These data allow for stating that consideration of sociodemographic characteristics of participants was a good decision, and that they should be controlled in the design and analysis of studies assessing AE.

The low loss rate – follow-up was completed in 99% of participants – was made possible by a comprehensive quality control of the whole process. The conduct of the study within the public healthcare system, used in Spain by the vast majority of the population, allowed for a reliable monitoring of pregnant women during pregnancy and the childbirth process, and for use of a preestablished internal communication network between the centres. Both these conditions greatly facilitated follow-up and control of the whole study process, resulting in a negligible loss rate.

## Competing interests

The authors declare that they have no competing interests.

## Authors' contributions

CPP and IAP conceived the idea for the study and they are the guarantors of the article. CPP, IAP, and GG led on the design of the study and obtained financial support. GG supervised field work, data management and statistical analysis. GRFG, IOH contributed to the development of the study protocol and acquired data related to delivery. IAP, ABH and JPG were responsible for the data management, quality control of the study process and contributed to data analysis. GMG and MUP contributed to the development of the study protocol and provided important advice regarding the measurement of personality and breastfeeding, respectively. All authors were responsible for the drafting of this paper, revised critically the article and approved the final version submitted to BMC Nursing.

## Pre-publication history

The pre-publication history for this paper can be accessed here:


